# Oleanolic Acid Diminishes Liquid Fructose-Induced Fatty Liver in Rats: Role of Modulation of Hepatic Sterol Regulatory Element-Binding Protein-1c-Mediated Expression of Genes Responsible for De Novo Fatty Acid Synthesis

**DOI:** 10.1155/2013/534084

**Published:** 2013-05-02

**Authors:** Changjin Liu, Ying Li, Guowei Zuo, Wenchun Xu, Huanqing Gao, Yifan Yang, Johji Yamahara, Jianwei Wang, Yuhao Li

**Affiliations:** ^1^Faculty of Basic Medical Sciences, Chongqing Medical University, 1 Yixueyuan Road, Yuzhong District, Chongqing 400016, China; ^2^College of Laboratory Medicine, Chongqing Medical University, Chongqing 400016, China; ^3^Endocrinology and Metabolism Group, Sydney Institute of Health Sciences/Sydney Institute of Traditional Chinese Medicine, Level 5, 545 Kent Street, Sydney, NSW 2000, Australia; ^4^Pharmafood Institute, Kyoto 602-8136, Japan; ^5^Department of Traditional Chinese Medicine, Chongqing Medical University, Chongqing 400016, China

## Abstract

Oleanolic acid (OA), contained in more than 1620 plants and as an aglycone precursor for naturally occurred and synthesized triterpenoid saponins, is used in China for liver disorders in humans. However, the underlying liver-protecting mechanisms remain largely unknown. Here, we found that treatment of rats with OA (25 mg/kg/day, gavage, once daily) over 10 weeks diminished liquid fructose-induced excess hepatic triglyceride accumulation without effect on total energy intake. Attenuation of the increased vacuolization and Oil Red O staining area was evident on histological examination of liver in OA-treated rats. Hepatic gene expression profile demonstrated that OA suppressed fructose-stimulated overexpression of sterol regulatory element-binding protein-(SREBP-) 1/1c mRNA and nuclear protein. In accord, overexpression of SREBP-1c-responsive genes responsible for fatty acid synthesis was also downregulated. In contrast, overexpressed nuclear protein of carbohydrate response element-binding protein and its target genes liver pyruvate kinase and microsomal triglyceride transfer protein were not altered. Additionally, OA did not affect expression of peroxisome proliferator-activated receptor-gamma- and -alpha and their target genes. It is concluded that modulation of hepatic SREBP-1c-mediated expression of the genes responsible for de novo fatty acid synthesis plays a pivotal role in OA-elicited diminishment of fructose-induced fatty liver in rats.

## 1. Introduction

Oleanolic acid (3*β*-hydroxyolean-12-en-28-oic acid), a pentacyclic triterpenoid compound, is contained in more than 146 families, 698 genera, and 1620 species of plants, including many foodstuffs (such as *Beta vulgaris* L., virgin olive oils), fruits (such as apple, pomegranate, and dates), and medicinal plants (such as *Crataegus pinnatifida Bunge*, *Aralia chinensis *L. var. nude, and *Eclipta alba*) [1–3]. Oleanolic acid not only occurs as a free acid, but also serves as an aglycone precursor for the triterpenoid saponins naturally occurred (such as ginsenosides and glycyrrhizic acid) and synthesized (such as 2-cyano-3,12-dioxooleana-1,9(11)-dien-28-oic acid (CDDO) and bardoxolone methyl) [1, 4, 5]. It has been well documented that oleanolic acid possesses many promising pharmacological activities, such as hepatoprotective, anti-inflammatory, antioxidant, and anticancer activities [1]. The hepatoprotective effects of oleanolic acid allow its use in China as an over-the-counter oral drug for liver disorders such as viral hepatitis in humans [1, 6–8]. Oleanolic acid and some naturally occurred and synthetic triterpenoids with the aglycone (such as glycyrrhizic acid and 2-cyano-3,12-dioxooleana-1,9-dien-28-oic-acid methyl ester) have also been demonstrated to improve abnormal lipid metabolism in rodents [1, 9–12]. We have also reported that pomegranate flower, which contains oleanolic acid [13], improves hypertriglyceridemia, excess cardiac lipid accumulation, and fatty liver in Zucker diabetic fatty rats [14, 15]. However, the underlying mechanisms of the hepatoprotective and lipid metabolism-regulating effects of oleanolic acid remain largely unknown.

Strong evidence suggests that chronic overconsumption of fructose results in fatty liver, dyslipidemia, insulin resistance, and obesity in animals and humans [16, 17]. Fatty liver (excessive accumulation of triglyceride in hepatocytes) is the hallmark of nonalcoholic fatty liver disease and nonalcoholic steatohepatitis, which has become an important public health problem due to its high prevalence, potential progression to severe liver disease, and association with cardiometabolic abnormalities [18, 19]. Despite the increasing incidence of nonalcoholic fatty liver disease and nonalcoholic steatohepatitis, there are no therapies currently approved for treatment of these common liver disorders.

In the present study, we tested the effects of oleanolic acid on fructose-induced fatty liver and investigated the underlying mechanisms in rats.

## 2. Materials and Methods

### 2.1. Animals, Diet, and Experimental Protocol

All animal procedures were in accordance with the “principles of laboratory animal care” (http://grants1.nih.gov/grants/olaw/references/phspol.htm) and were approved by the Animal Ethics Committee, Chongqing Medical University, China.

Male Sprague-Dawley rats weighing 210–230 g and the standard chow were supplied by the Laboratory Animal Center, Chongqing Medical University, China. Rats were housed in a temperature-controlled facility (21 ± 1°C, 55 ± 5% relative humidity) with a 12 h light/dark cycle. Animals were allowed free access to water and the standard chow for at least 1 week prior to starting the experiments.

It is known that sugar-sweetened nonalcoholic beverages, such as soft drinks, appear as the major source of fructose for all classes of age considered, except for children younger than 6 years and adults older than 50 years [17]. Thus, fructose in drinking water was used in the present study. In initial experiments, we found that compared to vehicle, oleanolic acid treatment significantly decreased fructose intake when the rats had free access to 10% fructose in drinking water. In order to exclude the influence of the variability in intake of fructose, the sole pathogenic factor in the development of the adverse metabolic effects in this model, the fructose consumption in oleanolic acid-treated rats was regulated to that of fructose controls. 24 rats were divided into 4 groups (*n* = 6 per group): (1) water control, free access to water; (2) fructose control, free access to 10% fructose solution (w/v, preparation every day); (3) fructose oleanolic acid 5 mg/kg, and (4) fructose oleanolic acid 25 mg/kg, in which the fructose concentration was adjusted once per 3 days based on the fructose consumption in the fructose control during the previous 3 days. There was no difference in body weight between the groups before treatments commenced. Animals in oleanolic acid-treated groups were administered oleanolic acid 5 or 25 mg/kg (Wako, Osaka, Japan, suspended in 5% Gum Arabic solution, gavage once daily) for 10 weeks, respectively. The rats in the corresponding water- and fructose-control groups received vehicle (5% Gum Arabic) alone. All rats had free access to the standard chow. To avoid stress and more accurately monitor fructose intake, 2 rats were housed in a cage. The consumed chow and fructose solution were measured per 2 rats daily and the intake of fructose was calculated. On day 70, rats were deprived of chow, but still had free access to water (Group 1) or fructose solution (Groups 2–4) overnight. Blood samples were collected by retroorbital venous puncture under ether anesthesia at 9:00–12:00 a.m. for determination of plasma concentrations of total cholesterol (kit from Kexin Institute of Biotechnology, Shanghai, China), triglyceride (Triglyceride-E kit, Wako, Osaka, Japan), nonesterified fatty acid (NEFA) (NEFA-C kit, Wako, Osaka, Japan), glucose (kit from Kexin Institute of Biotechnology, Shanghai, China), and insulin (kit from Morinaga Biochemical Industries, Tokyo, Japan). Immediately, animals were weighed and killed by prompt dislocation of the neck vertebra. Livers, epididymal and peri-renal adipose tissues (e + p WAT) were collected and weighed, respectively. The ratios of liver weight and e + p WAT weight to body weight were calculated. Segments of liver were snap frozen in liquid nitrogen and stored at −80°C for subsequent determination of gene/protein expression and triglyceride contents.

### 2.2. Determination of Triglyceride Content in Liver

Triglyceride content in liver was determined as described previously [20]. Briefly, 100 mg of tissue was homogenized and extracted with 2 mL of isopropanol. After centrifugation (3000 rpm), the triglyceride content in supernatants was determined enzymatically (Wako, Osaka, Japan).

### 2.3. Histological Examination

A portion of liver was fixed with 10% formalin and embedded in paraffin. Three-micron sections were cut and stained with hematoxylin and eosin for examination of liver histology (BX-51, Olympus Corporation, Tokyo, Japan). To further confirm lipid droplet accumulation, six-micron frozen sections were stained with Oil Red O. Forty fields in three individual sections were randomly selected, and the Oil Red O-stained area and the total tissue area were measured using an ImageJ 1.43 analyzing system. The ratio of the Oil Red O-stained area to the total tissue area was calculated (%). 

### 2.4. Real-Time PCR

Real-time PCR was performed as described previously [21, 22]. Total RNA was isolated from livers of individual rats using TRIzol (Takara, Dalian, China). cDNA was synthesized using M-MLV RTase cDNA Synthesis Kit (Takara, Dalian, China) according to the manufacturer's instructions. Real-time PCR was performed with the CFX 96 Real-Time PCR Detection System (Biorad Laboratories Inc., Hercules, CA, USA) using the SYBR Premix Ex Taq II (Takara, Dalian, China). The sequences of primers are shown in Table 1. The gene expression from each sample was analysed in duplicates and normalized against the internal control gene *β*-actin. Levels in water control rats were arbitrarily assigned a value of 1.

### 2.5. Western Blot

Western blot was performed as described previously [21]. Nuclear protein was prepared individually from livers using the NE-PER nuclear and cytoplasmic extraction reagent kit (Pierce Biotechnology, Rockford, IL, USA), according to the manufacturer's instructions. Protein concentration was determined using the Bradford method (Bio Rad Laboratories, Hercules, CA, USA) using bovine serum albumin as a standard. Nuclear protein (30 *μ*g) was subjected to SDS-PAGE analysis on a 10% gel. Proteins were electrotransferred onto Polyvinylidene Fluoride Membrane (Amersham, Buckinghamshire, UK). Sterol regulatory element-binding protein- (SREBP-) 1 (dilution 1 : 200, Santa Cruz Biotechnology, Santa Cruz, CA, USA) and carbohydrate response element-binding protein (ChREBP) (dilution 1 : 1000, Novus Biologicals, Littleton, CO, USA) were detected with a goat polyclonal antibody and rabbit polyclonal antibody, respectively. Detection of signals was performed using the ECL Western blot detection kit (Pierce Biotechnology, Rockford, IL, USA) with anti-goat and anti-rabbit horseradish peroxidase-conjugated IgG (dilution 1 : 5,000, Santa Cruz Biotechnology, Santa Cruz, CA, USA) as second antibody, respectively. Polyclonal rabbit lamin A/C antibody (dilution 1 : 1000, Cell Signaling Technologies, Beverly, MA, USA) was used as loading control to normalize the signals obtained for nuclear SREBP-1 and ChREBP proteins. The immunoreactive bands were visualized by autoradiography and the density was evaluated using ImageJ 1.43. Levels in water control rats were arbitrarily assigned a value of 1.

### 2.6. Data Analysis

All results are expressed as means ± SEM. Data were analyzed by ANOVA using the StatView software, and followed by The Student-Newman-Keuls test to locate the differences between groups. *P* < 0.05 was considered to be statistically significant.

## 3. Results

### 3.1. Effects on Fructose-Induced Adverse Effects in Rats

**Figure 1:**
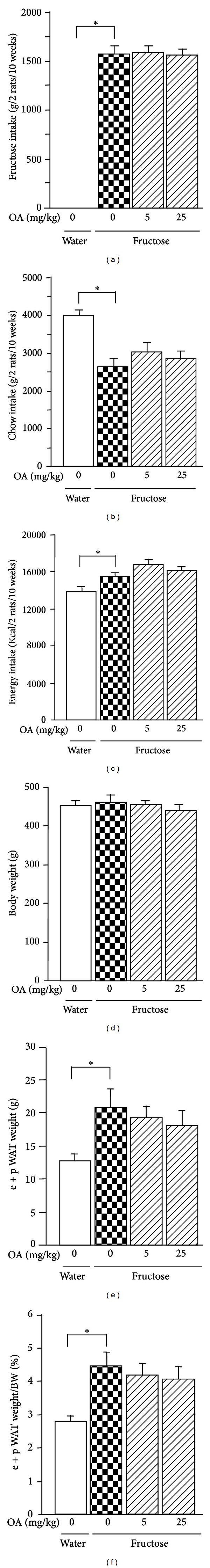
Intakes of fructose (a), laboratory chow (b), and energy (c), body weight (d), epididymal and perirenal white adipose tissue (e + p WAT) weight (e), and the ratio of e + p WAT weight to body weight (f) in water-control, 10% fructose solution-control and fructose pair-fed oleanolic acid- (OA-) treated rats. Animals were administered with OA (5 or 25 mg/kg/day) or vehicle (OA: 0 mg/kg, 5% Gum Arabic) by oral gavage daily for 10 weeks. Data are means ± SEM (*n* = 6 each group). *P < 0.05.

Intake of 10% fructose solution decreased the intake of chow (Figure 1(a)), but the total energy intake was increased (Figure 1(c)), compared to water drinking. Although fructose feeding did not affect body weight (Figure 1(d)), e + p WAT weight (Figure 1(e)) and the ratio of e + p WAT weight to body weight (Figure 1(f)) were increased. After fructose intake was uniform in fructose control and oleanolic acid-treated groups (Figure 1(a)), treatment with oleanolic acid did not show significant effect on chow (Figure 1(b)) and energy (Figure 1(c)) intakes, body weight (Figure 1(d)), e + p WAT weight (Figure 1(e)), and the ratio of e + p WAT weight to body weight (Figure 1(f)).

**Figure 2:**
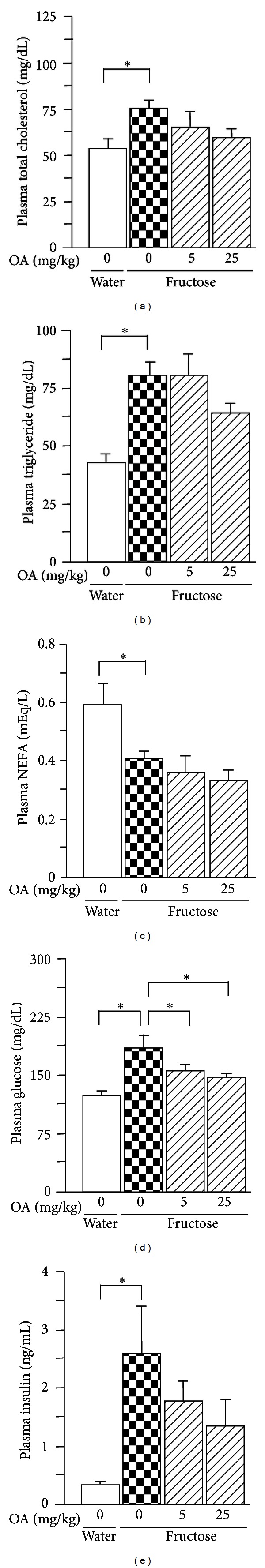
Plasma total cholesterol (a), triglyceride (b), NEFA (c), glucose (d), and insulin (e) concentrations in water-control, 10% fructose solution-control, and fructose pair-fed oleanolic acid- (OA-) treated rats. Animals were administered with OA (5 or 25 mg/kg/day) or vehicle (OA: 0 mg/kg, 5% Gum Arabic) by oral gav age daily for 10 weeks. Data are means ± SEM (*n* = 6 each group). *P < 0.05.

Under the status of feeding fructose solution but deprival of chow supply, plasma concentrations of total cholesterol (Figure 2(a)), triglyceride (Figure 2(b)), glucose (Figure 2(d)), and insulin (Figure 2(e)) were elevated, whereas plasma NEFA concentration (Figure 2(c)) was lower, compared to those of water feeding group. Oleanolic acid treatment decreased plasma glucose concentration (Figure 2(d)) and had a trend to suppress the increase in plasma insulin concentration (Figure 2(e)). The differences in plasma concentrations of total cholesterol (Figure 2(a)), triglyceride (Figure 2(b)), and NEFA between fructose control and oleanolic acid-treated groups were without statistic significance.

**Figure 3:**
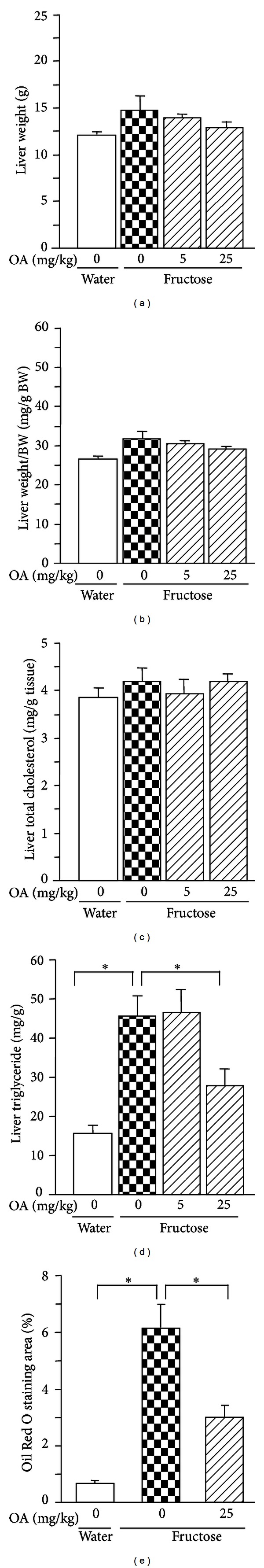
Liver weight (a), the ratio of liver weight to body weight (b), liver total cholesterol content (c), liver triglyceride content (d), and Oil Red O staining area (e) in water-control, 10% fructose solution-control and fructose pair-fed oleanolic acid- (OA-) treated rats. Animals were administered with OA (5 or 25 mg/kg/day) or vehicle (OA: 0 mg/kg, 5% Gum Arabic) by oral gavage daily for 10 weeks. Data are means ± SEM (*n* = 6 each group). *P < 0.05.

**Figure 4:**
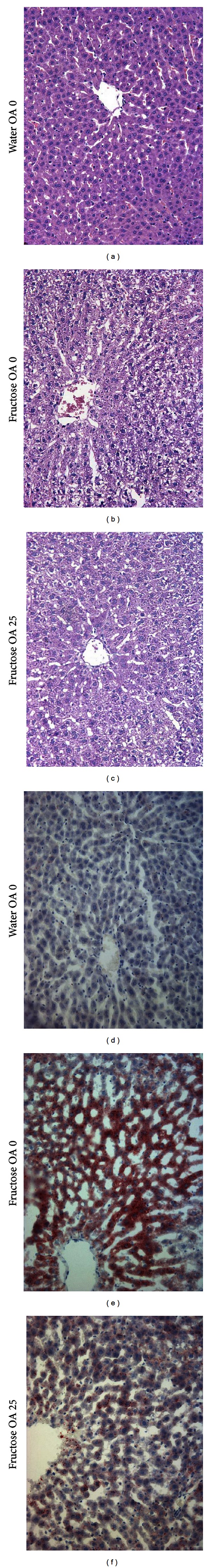
Representative images showing histology of liver (hematoxylin and eosin staining (a)–(c); Oil Red O staining (d)–(f) ×200) in water-control, 10% fructose solution-control, and fructose pair-fed oleanolic acid- (OA-) treated rats at week 10. Animals were administered with OA (25 mg/kg/day) or vehicle (OA: 0 mg/kg, 5% Gum Arabic) by oral gavage daily for 10 weeks. Data are means ± SEM (*n* = 6 each group). *P < 0.05.

Although fructose feeding did not significantly affect liver weight (Figure 3(a)), the ratio of liver weight to body weight (Figure 3(b)), and hepatic total cholesterol content (Figure 3(c)), it increased triglyceride content to 3-folds of that in water control group (Figure 3(d)). In accord with this finding, increased vacuolization (Figure 4(b)) and Oil Red O staining area (Figures 3(e) and 4(e)) were evident on histological examination of liver sections from fructose-fed rats compared with water-control rats (Figures 3(d), 4(a), and 4(d)), indicative of excess lipid droplet accumulation. Oleanolic acid treatment at 25 mg/kg decreased hepatic triglyceride content (Figure 3(d)). Consistently, vacuolization (Figure 4(c)) and Oil Red O staining area (Figures 3(e) and 4(f)) in liver were also reduced. Low dosage (5 mg/kg) showed no effect on these variables. Oleanolic acid treatment did not alter liver weight (Figure 3(a)), the ratio of liver weight to body weight (Figure 3(b)), and hepatic total cholesterol content (Figure 3(c)).

### 3.2. Hepatic Gene/Protein Expression Profiles in Rats

**Figure 5:**
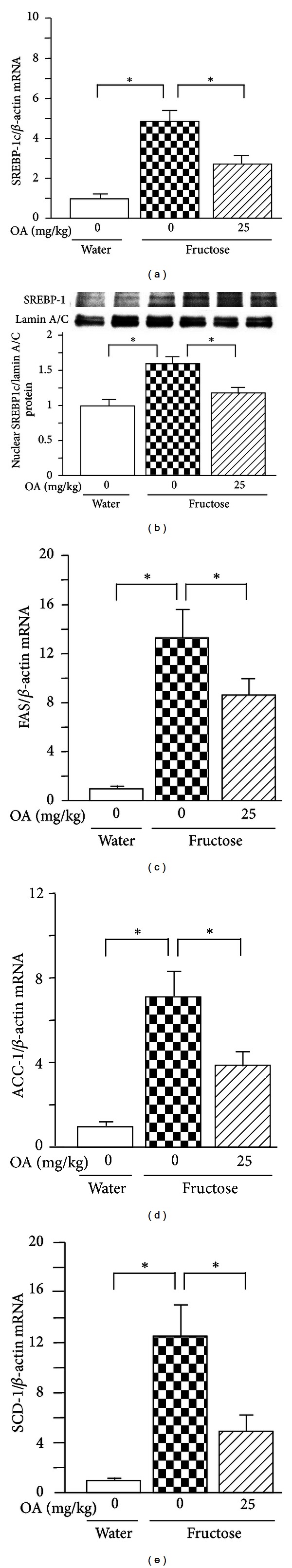
Hepatic expression of mRNAs encoding sterol regulatory element-binding protein- (SREBP-) 1c (a), fatty acid synthase (FAS) (c), acetyl-CoA carboxylase- (ACC-) 1 (d) and stearoyl-CoA desaturase- (SCD-) 1 (e), and nuclear SREBP-1 protein (b) in water-control, 10% fructose solution-control, and fructose pair-fed oleanolic acid- (OA-) treated rats at week 10. Animals were administered with OA (25 mg/kg/day) or vehicle (OA: 0 mg/kg, 5% Gum Arabic) by oral gavage daily for 10 weeks. mRNA was determined by real-time PCR. Protein expression was determined by Western blot. Data are means ± SEM (*n* = 6 each group). *P < 0.05.

**Figure 6:**
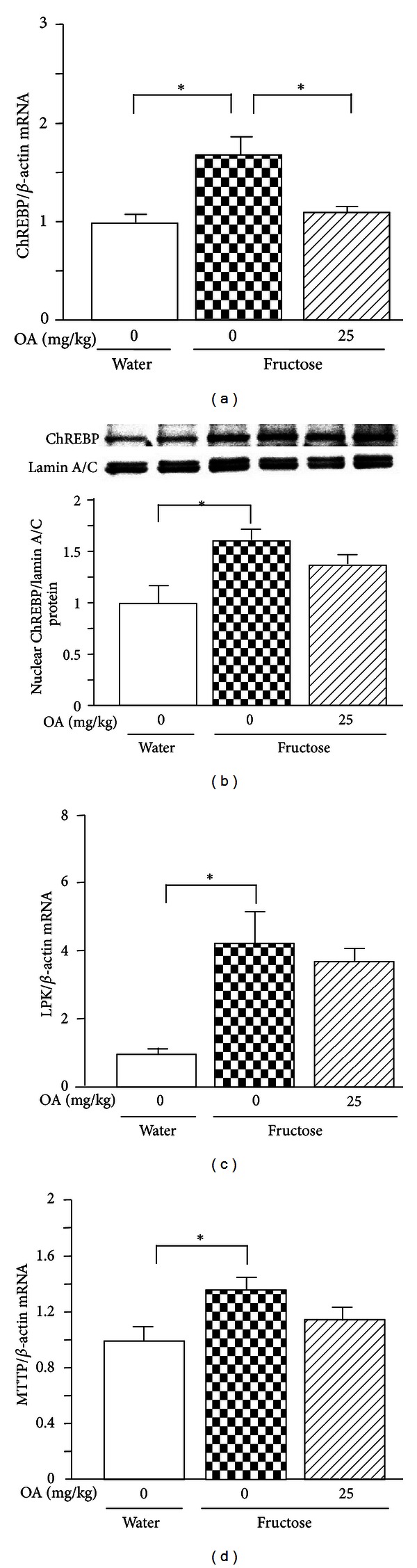
Hepatic expression of mRNAs encoding carbohydrate response element-binding protein (ChREBP) (a), liver pyruvate kinase (LPK) (c), and microsomal triglyceride transfer protein (MTTP) (d), and nuclear ChREBP protein (b) in water-control, 10% fructose solution-control, and fructose pair-fed oleanolic acid- (OA-) treated rats at week 10. Animals were administered with OA (25 mg/kg/day) or vehicle (OA: 0 mg/kg, 5% Gum Arabic) by oral gavage daily for 10 weeks. mRNA was determined by real-time PCR. Protein expression was determined by Western blot. Data are means ± SEM (*n* = 6 each group). *P < 0.05.

By real-time PCR fructose feeding substantially increased mRNA levels of SREBP-1c (Figure 5(a)), fatty acid synthase (FAS) (Figure 5(c)), acetyl-CoA carboxylase- (ACC-) 1 (Figure 5(d)), stearoyl-CoA desaturase- (SCD-) 1 (Figure 5(e)), ChREBP (Figure 6(a)), liver pyruvate kinase (LPK) (Figure 6(c)), and microsomal triglyceride transfer protein (MTTP) (Figure 6(d)). The increase in nuclear SREBP-1 and ChREBP protein content was demonstrated by Western blot analysis (Figures 5(b) and 6(b)). After oleanolic acid treatment (25 mg/kg), pronounced suppression of mRNAs encoding SREBP-1c (Figure 5(a)), FAS (Figure 5(c)), ACC-1 (Figure 5(d)), and SCD-1 (Figure 5(e)) was noted. The results of nuclear protein expression further confirmed the suppression of SREBP-1 by oleanolic acid treatment (Figure 5(b)). Although oleanolic acid treatment also decreased ChREBP mRNA level (Figure 6(a)), it did not significantly alter nuclear ChREBP protein content (Figure 6(b)). Consistent with the finding in nuclear ChREBP protein expression, the target genes LPK (Figure 6(c)) and MTTP (Figure 6(d)) mRNA levels were also without significant change.

**Figure 7:**
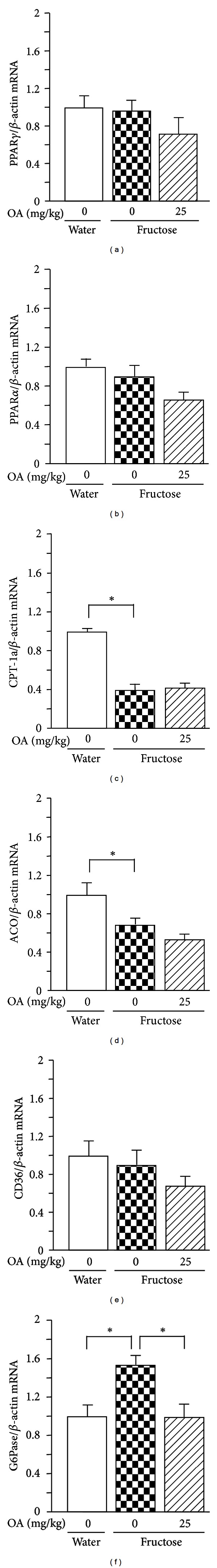
Hepatic expression of mRNAs encoding peroxisome proliferator-activated receptor-(PPAR-) *γ* (a), PPAR-*α* (b), carnitine palmitoyltransferase- (CPT-) 1a (c), acyl-CoA oxidase (ACO) (d), CD36 (e), and glucose-6-phosphatase (G6Pase) (f) in water-control, 10% fructose solution-control and fructose pair-fed oleanolic acid- (OA-)treated rats at week 10. Animals were administered with OA (25 mg/kg/day) or vehicle (OA: 0 mg/kg, 5% Gum Arabic) by oral gavage daily for 10 weeks. mRNA was determined by real-time PCR. Data are means ± SEM (*n* = 6 each group). *P < 0.05.

Fructose feeding did not alter mRNA levels of peroxisome proliferator-activated receptor- (PPAR-) *γ* (Figure 7(a)), PPAR-*α* (Figure 7(b)), and CD36 (Figure 7(e)), whereas expressions of carnitine palmitoyltransferase- (CPT-) 1a (Figure 7(c)), and acyl-CoA oxidase (ACO) (Figure 7(d)) were decreased. In contrast, glucose-6-phosphatase (G6Pase) was upregulated by fructose feeding (Figure 7(f)). Oleanolic acid treatment suppressed the increased G6Pase gene expression, but was without effect on mRNA levels of other genes (Figures 7(a)–7(f)).

## 4. Discussion

Although the molecular mechanism leading to the development of hepatic steatosis in the pathogenesis of nonalcoholic fatty liver disease is complex, animal models have shown that modulating important enzymes, such as ACC, FAS, and SCD-1, in fatty acid synthesis in liver may be a key for the treatment of nonalcoholic fatty liver disease [19]. Fructose, by providing large amounts of hepatic triose-phosphate as precursors for fatty acid synthesis, is highly lipogenic [17]. Recent studies in humans and in rodents have demonstrated that an increase in de novo hepatic lipogenesis plays a pivotal role in fructose feeding-induced excessive hepatic fat accumulation [17, 23]. In the present study, oleanolic acid treatment diminished long-term liquid fructose overconsumption-induced excess lipid accumulation in the liver of rats without effect on intakes of chow and total energy, body weight, and epididymal and perirenal white adipose weight. However, fructose feeding-stimulated overexpression of ACC-1, FAS, and SCD-1 was substantially suppressed by oleanolic acid treatment. Thus, these results suggest that oleanolic treatment diminishes fructose-induced fatty liver via modulation of hepatic genes responsible for de novo fatty acid synthesis.

SREBP-1c, the principal inducer of hepatic lipogenesis [17, 19], is responsible for the insulin-mediated induction of lipogenic enzymes, such as ACC, FAS, and SCD-1, in the liver [19]. However, SREBP-1c may be expressed independently of insulin in fructose metabolism [24]. Dietary fructose activates lipogenesis partly through inducing expression of SREBP-1 in rats [25]. A genetic polymorphism within the SREBP-1c promoter region among strains of inbred mice underlies a differential sensitivity to fructose-induced lipid synthesis, also suggesting that this transcription factor mediates the lipogenic effects of fructose [26]. In the present study, fructose feeding stimulated hepatic expression of SREBP-1/1c at mRNA and nuclear protein levels in rats. Treatment with oleanolic acid suppressed the overexpression of both SREBP-1c mRNA and nuclear SREBP-1 protein. Thus, these findings suggest that modulation of the SREBP-1c-mediated pathway is responsible for oleanolic acid-elicited improvement of fructose-induced fatty liver.

It has been suggested that the lipogenic genes require both ChREBP and SREBP-1c to be fully activated [27, 28]. It is known that a high-fructose diet stimulates SREBP-1c and ChREBP through currently unknown mechanisms [17]. ChREBP has emerged as a major mediator of glucose action on lipogenic gene expression and as a key determinant of lipid synthesis in vivo [19, 29]. Although ChREBP is localized in the cytosol, the endogenous ChREBP protein is addressed into the nucleus in response to high glucose concentrations in primary hepatocytes and in response to a high-carbohydrate diet in liver of mice [29]. Thus, ChREBP localization in the nucleus of cells is a key determinant of its functional activity [29]. Activation of ChREBP acts in synergy with SREBP to increase the expression of the lipogenic genes [17, 30]. ChREBP is responsible for glucose-induced transcription of LPK [31, 32], while LPK is not subjected to SREBP-1c regulation [33]. ChREBP also works together with its functional partner Max-like protein X on carbohydrate-response elements to regulate MTTP, a protein that catalyzes the rate-limiting step in the production of apoB-containing very low-density lipoprotein [34]. It has been demonstrated that the decrease in hepatic expression of ChREBP with a specific antisense oligonucleotide results in downregulation of hepatic expression of MTTP, accompanied by a decrease in plasma triglyceride concentration in fructose-fed rats [35]. In the present study, fructose feeding also simultaneously stimulated hepatic expression of ChREBP at mRNA and nuclear protein levels in rats, accompanied by upregulation of LPK and MTTP. We have recently demonstrated that ginger extract treatment suppressed fructose-induced overexpression of hepatic ChREBP with minimal effect on SREBP1c in rats [21]. It is interesting in the present study that although ChREBP mRNA was decreased, the nuclear ChREBP protein level was not altered significantly after oleanolic acid treatment. Further, overexpressed LPK and MTTP mRNAs and hypertriglyceridemia were also without significant change. Thus, these findings suggest that the ChREBP-mediated pathway may play a less role in the effects of oleanolic acid.

PPAR-*α* and -*γ* are members of the ligand-activated nuclear receptor superfamily and predominantly expressed in liver and adipose tissue, respectively [36]. Pharmacological activation of PPAR-*γ* upregulates the genes encoding molecules that promote a combination of lipid storage and lipogenesis, such as CD36, SREBP-1, and SCD-1 [36]. Activation of this metabolic pathway causes bodywide lipid repartitioning by increasing the triglyceride content of adipose tissue and lowering free fatty acids and triglycerides in the circulation, liver, and muscle, thereby improving insulin sensitivity [36]. In contrast, PPAR-*α* influences intracellular lipid and carbohydrate metabolism through direct transcriptional control of the genes involved in peroxisomal and mitochondrial *β*-oxidation pathways, fatty, acid uptake and triglyceride catabolism, such as CPT-1a, ACO, and also CD36 [36]. It has been reported that oleanolic acid enhances PPAR-*α* expression and activities in human embryonic kidney 293 cells [14], CV-1 monkey kidney cells, and human skin keratinocyte cell line HaCaT [37, 38]. Furthermore, oleanolic acid was also found to have human PPAR-*γ* ligand-binding activity in CV-1 monkey kidney cells using a GAL4-PPAR-*γ* chimera assay [39]. In the present study, 10-week liquid fructose feeding downregulated CPT-1a and ACO mRNAs in the livers of rats. The results were consistent with the previous finding that fructose feeding reduces hepatic PPAR-*α* activity [40]. However, oleanolic acid treatment was without effect on hepatic expression of PPAR-*α*, CPT-1a, and ACO. On the other hand, hepatic expression of PPAR-*γ* and CD36 was unchanged, whereas the overexpressed SREBP-1c and SCD-1 were substantially downregulated by oleanolic acid treatment. Thus, our findings in gene expression do not support the involvement of the hepatic PPAR-*α* and -*γ* pathways in the antisteatotic effect of oleanolic acid treatment. It is needed to investigate whether the differences in conditions (in vivo versus in vitro) and/or cell species (rat hepatocyte versus human embryonic kidney 293 cells, CV-1 monkey kidney cell, or human skin keratinocyte cell) induce the discrepancy in PPAR-*α*- and -*γ*-mediated gene expression. Further studies are also needed to investigate whether oleanolic acid modulates adipose PPAR-*γ*-mediated gene expression and activities in fructose-fed rats.

The deleterious effect of fructose on glucose metabolism and insulin sensitivity has been demonstrated in healthy men and rodents [17]. It is well known that excessive accumulation of triglyceride in hepatocytes is strongly associated with hepatic or systemic insulin resistance (lipotoxicity) [19]. In contrast, decreasing hepatic triglyceride pools correlates with improved insulin sensitivity [19]. In the present study, oleanolic acid treatment attenuated fructose-induced increase in plasma glucose concentration and had a trend to ameliorate the hyperinsulinemia in rats. Moreover, oleanolic acid suppressed fructose-stimulated upregulation of hepatic expression of G6Pase, one of the important gluconeogenic enzymes. Thus, these results suggest that oleanolic acid treatment ameliorates fructose overconsumption-induced insulin resistance, which is possibly associated with improvement of fatty liver. 

In conclusion, our present results demonstrate that treatment with oleanolic acid ameliorates fructose-induced fatty liver in rats, in which modulation of the hepatic SREBP-1c-mediated pathway plays a pivotal role. Our findings may provide better understanding of oleanolic acid and associated chemicals and herbs in the treatment of liver disorders, especially nonalcoholic fatty liver disease.

## Figures and Tables

**Table 1:** Primer sequences for real-time PCR assays.

Gene	Forward primers	Reverse primers
*β*-Actin	ACGGTCAGGTCATCACTATCG	GGCATAGAGGTCTTTACGGATG
ACC-1	AACATCCCGCACCTTCTTCTAC	CTTCCACAAACCAGCGTCTC
ACO	CCCAAGACCCAAGAGTTCATTC	TCACGGATAGGGACAACAAAGG
CD36	AACCCAGAGGAAGTGGCAAAG	GACAGTGAAGGCTCAAAGATGG
ChREBP	GAAGACCCAAAGACCAAGATGC	TCTGACAACAAAGCAGGAGGTG
CPT-1a	CTGCTGTATCGTCGCACATTAG	GTTGGATGGTGTCTGTCTCTTCC
FAS	ACCTCATCACTAGAAGCCACCAG	GTGGTACTTGGCCTTGGGTTTA
G6Pase	GAGTGGCTCAACCTCGTCTTC	AAGGGAACTGGTGAATCTGGAC
LPK	GACCCGAAGTTCCAGACAAGG	ATGAGCCCGTCGTCAATGTAG
MTTP	CAAAGTATGAAAGGCTATCCACAGG	GGAATACCACGTTGCACATCTCA
PPAR-*α*	GTCATCACAGACACCCTCTCCC	TGTCCCCACATATTCGACACTC
PPAR-*γ*	GCCCTTTGGTGACTTTATGGAG	GCAGCAGGTTGTCTTGGATGT
SCD-1	CAGTTCCTACACGACCACCACTA	GGACGGATGTCTTCTTCCAGAT
SREBP-1c	CTGTCGTCTACCATAAGCTGCAC	ATAGCATCTCCTGCACACTCAGC

Sequences: 5′ to 3′.

## Abbreviations

ACC: Acetyl-CoA carboxylase
ACO: Acyl-CoA oxidase
ChREBP: Carbohydrate response element binding protein
CPT: Carnitine palmitoyltransferase
FAS: Fatty acid synthase
G6Pase: Glucose-6-phosphatase
LPK: Liver pyruvate kinase
MTTP: Microsomal triglyceride transfer protein
NEFA: Nonesterified fatty acids
PPAR: Peroxisome proliferator-activated receptor
SCD: Stearoyl-CoA desaturase
SREBP: Sterol regulatory element-binding protein
WAT: White adipose tissue.
